# Automatic MTF Conversion between Different Characteristics Caused by Imaging Devices

**DOI:** 10.3390/jimaging10020049

**Published:** 2024-02-17

**Authors:** Midori Tanaka, Tsubasa Ando, Takahiko Horiuchi

**Affiliations:** 1 Graduate School of Global and Transdisciplinary Studies, Chiba University, Yayoi-cho 1-33, Inage-ku, Chiba 263-8522, Japan; 2 Graduate School of Science and Engineering, Chiba University, Yayoi-cho 1-33, Inage-ku, Chiba 263-8522, Japanhoriuchi@faculty.chiba-u.jp (T.H.)

**Keywords:** MTF, measurement of material appearance, appearance control, image quality improvement

## Abstract

Depending on various design conditions, including optics and circuit design, the image-forming characteristics of the modulated transfer function (MTF), which affect the spatial resolution of a digital image, may vary among image channels within or between imaging devices. In this study, we propose a method for automatically converting the MTF to the target MTF, focusing on adjusting the MTF characteristics that affect the signals of different image channels within and between different image devices. The experimental results of MTF conversion using the proposed method for multiple image channels with different MTF characteristics indicated that the proposed method could produce sharper images by moving the source MTF of each channel closer to a target MTF with a higher MTF value. This study is expected to contribute to technological advancements in various imaging devices as follows: (1) Even if the imaging characteristics of the hardware are unknown, the MTF can be converted to the target MTF using the image after it is captured. (2) As any MTF can be converted into a target, image simulation for conversion to a different MTF is possible. (3) It is possible to generate high-definition images, thereby meeting the requirements of various industrial and research fields in which high-definition images are required.

## 1. Introduction

In imaging devices, it is important to take into account the optical characteristics of image acquisition, processing, and generation during capturing, developing, and forming images. This is essential to ensure the clear and accurate communication of the optical information of the real object. Although significant progress has been made in optical design, considering aspects such as light scattering and aberrations [1,2], challenges persist when dealing with optical information in consumer imaging devices subject to design restrictions on cost and miniaturization. Aberrations, including distortion and blur, become inevitable in such scenarios, leading to a range of issues such as reduced image clarity, false colors, and the occurrence of artifacts. Furthermore, the appearance and perceived quality of a reproduced image may differ from those of the real object [3]. Therefore, when images acquired by imaging devices such as cameras and scanners are generated for display on a screen or projector, or printed on paper, the image quality is generally improved by applying image-processing techniques such as sharpening filters.

The modulation transfer function (MTF) has long been used as an index for evaluating the performance of optical systems such as lenses and cameras. The MTF indicates the contrast modulation characteristics of images in the frequency domain, and its metric is specified in ISO 12233 [4]. Characteristics of camera lenses and sensors, such as light scattering and aberrations, can change the MTF and cause image blurring. Conventional studies have focused on the accuracy [5,6] and simplicity [7] of the MTF calculation method. Several previous studies have attempted to increase the MTF by improving optical systems, such as lenses and circuits, for applications that require high-resolution images, such as remote sensing and aerospace applications that handle satellite images [8,9,10]. Developing techniques to maintain a high MTF is important, particularly in fields requiring high-quality images (e.g., medical imaging, security cameras, and remote sensing). To improve image blurring caused by spatially different image formation characteristics in the image plane of the captured image, it is common to utilize the image formation characteristic information (point spread function and MTF) of the image sensor or to convert pixel values by filter processing using known image formation characteristics based on optical shooting conditions. These image formation characteristics are difficult to implement without the manufacturer of the camera or image sensor [11,12] and cannot be handled by end users.

A software-based image-sharpening process is a possible method for improving image blurring, even when optical information is unknown. Typical methods include sharpening filters, unsharp masking, edge detection algorithms, high-pass filters, and methods based on deep learning, which are important for image processing [13,14,15,16,17,18]. Recent trends have focused on machine-learning-based methods, such as deep neural networks, for image quality evaluation and improvement [19,20]. These approaches to image quality improvement through image processing have enabled high-quality sharpening and improved the visual appearance of the detailed texture and contour information in images. Image sharpening by image processing, such as unsharp masking, is widely used to improve image blurring, in which the converted image is visually and arbitrarily enhanced and can be converted into an optically unnatural image, increasing noise and creating artifacts owing to excessive sharpening and local edge enhancement. In addition, appropriate parameter settings are required to extract fine details from the image. Another issue is the development of generic algorithms to handle different image types and qualities. It is also difficult to find a generic approach, because the optimal sharpening method may vary depending on the quality and blurring level of the original image.

To the best of our knowledge, no studies have been conducted to improve the MTF for end users. In our trial, we attempted to control the MTF by sharpening the filters of the three channels of the color camera [21]. However, the MTF conversion between channels using sharpening filters is far from the MTF of common camera lenses, and the optical certainty of the MTF is questionable. We have also worked on managing the total appearance of digital images generated by different imaging devices [22,23]. Although we converted the perceptual glossiness, transparency, and other qualities perceived from the reproduced image, we were not able to control frequency information, such as MTF.

Generally, widely used imaging devices have different image characteristics owing to the different designs of each device. Therefore, when comparing images acquired by multiple imaging devices, different image information is acquired and generated even though the target object is the same. In this study, we propose a method to convert the MTFs of imaging devices to be handled closer to the target MTF in order to match the optical characteristics that differ between images. Specifically, the spatial modulation characteristic MTF, which represents the image formation characteristics, is used to match the target MTF for multiple image channels with different image formation characteristics within or between imaging devices. Here, conversion means not only the improvement of image blur caused by low MTF (conversion to boost MTF) but also the generation of image blur (conversion to lower MTF) and adjusts the MTF of the actual image device, which varies depending on the optical characteristics, such as wavelength, filters, and other physical imaging device characteristics. The derived conversion relationship can be applied to a variety of images and can output images with MTF matching between channels. 

Our motivation for this study is to develop an appearance management technology to reduce differences in resolution characteristics among imaging devices, as color management technology controls colors to reduce color differences among imaging devices and to improve the appearance of images. The approach of converting MTFs between image channels is novel and not found in existing studies and has the potential to contribute to the development of various industrial technologies, such as reducing prototype costs through the application of image simulation technology in the design of imaging equipment. The contributions and novelty of this study are as follows: (1) to reduce differences in the appearance of generated images caused by characteristic differences between imaging devices; (2) to reduce the impact of aberrations in the design of imaging devices on the appearance of images, which is an orthodox issue in imaging; and (3) to make MTF-based image simulation possible by generating the spatial resolution of image variables through MTF conversion.

## 2. Methods

In this section, we propose a method for converting the MTF closer to the target MTF for multiple image channels with different imaging characteristics within or between imaging devices.

### 2.1. Overall Procedure

**Figure 1:**
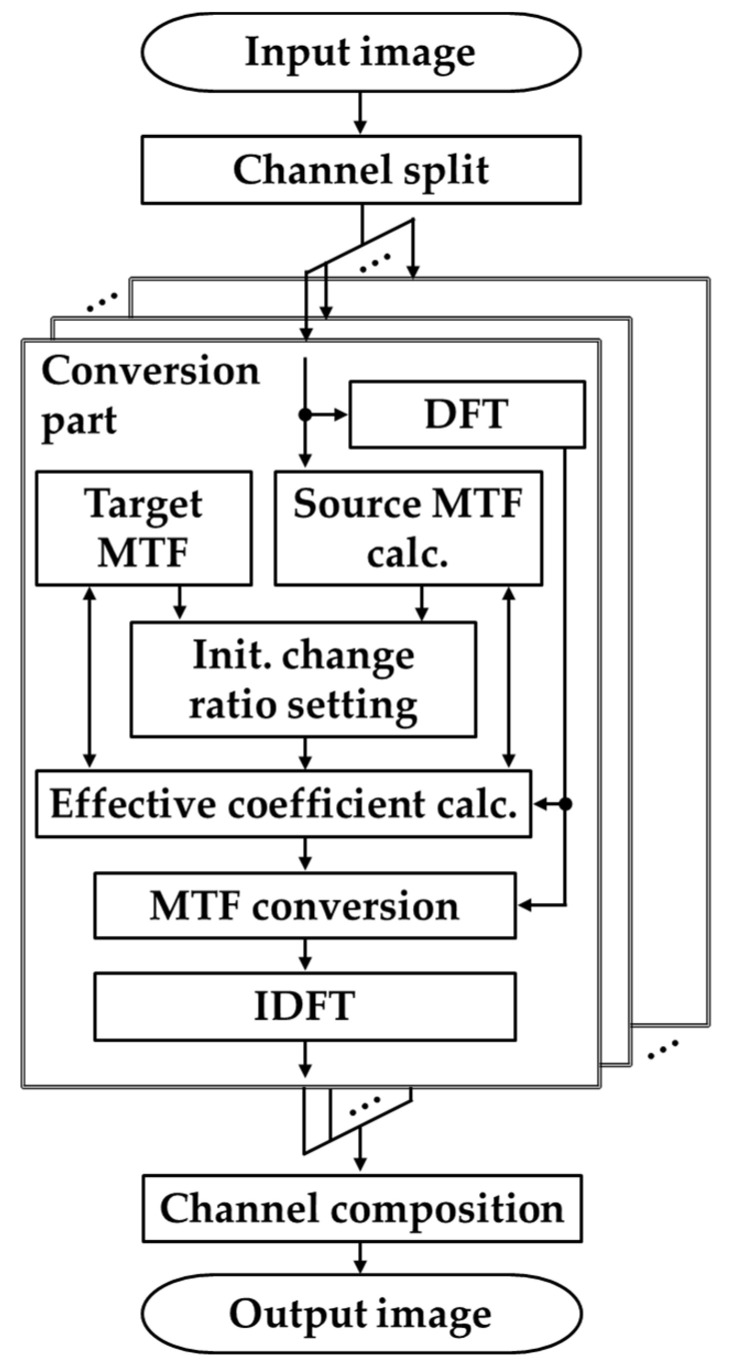
Flowchart of the proposed method.

The MTF conversion process between multiple channel images is described based on the flow of the proposed method, as shown in Figure 1. In the proposed method, for multiple channels (e.g., R, G, and B channels), in which the input image is divided into each channel image, MTF conversion is performed on each channel image according to the process shown in the conversion section, and each channel image after conversion is combined to generate the output image. At the beginning and end of the conversion, 2D discrete Fourier transform (DFT) and 2D discrete inverse Fourier transform (IDFT) are performed on the input image to modulate the MTF conversion in the frequency space. Because the proposed method is more efficient for processing in the frequency space, DFT is applied to represent the image as a spectrum in the frequency space, and frequency modulation is applied to transform the MTF for each frequency in the frequency space. Finally, the modulated frequency information is converted back into image information using IDFT.

In the conversion part, the proposed MTF conversion is performed using the source MTF, target MTF, single-channel image, and its frequency information. Here, the target MTF is the desired MTF obtained through measurement or simulation using an existing technique. It is theoretically possible to have a target MTF that is uniform across channels or that is individually different. However, as discussed in the main body, it is undesirable to have different MTFs across channels from a practical perspective because it leads to problems such as purple fringing. Therefore, it is desirable to have a uniform MTF across the channels. The source MTF was calculated from the input image by measurements using existing methods (e.g., [4]). Using these, the initial change ratio set and effective coefficient calculations were performed to bring the source MTF close to the target MTF, and amplification and damping conversions were performed for each frequency of the source MTF in the MTF conversion. Specifically, the input image spectrum and coefficient candidates are multiplied in the frequency space in sequence, and the effective coefficients, that is, the optimal coefficients, are determined to bring the source MTF of the channel image obtained by IDFT close to the target MTF.

### 2.2. MTF Conversion

The conversion of the source MTF was implemented by generating a coefficient array of the same size as the number of vertical and horizontal pixels in the input image and multiplying it by the input image spectrum in the frequency space.

In this study, the MTF frequency is expressed in cycles per pixel (cpp). In other words, the Nyquist frequency of the cpp in an image is 0.5 cpp. This is because the best representation of frequency in an image is when two pixels are used to represent one cycle. In other words, the maximum frequency occurs when one cycle is represented by two pixels. Thus, the Nyquist frequency becomes 0.5 cpp. Therefore, the proposed method modulates the frequency range by up to 0.5 cpp. First, the frequencies in the range from 0 cpp to 0.5 cpp are quantized to *N* points. Let f[i] cpp and m[i] be the i-th frequency obtained from the DC component (i = 1) and its MTF value, respectively. Then, the change ratio r[i] for f[i−1] at frequency f[i] is defined by the following equation:

(1) $$r\left[i\right]=c\left[i\right]/c[i-1],\;i=2,\ldots ,N$$

where *c*$\left[i\right]$ is the coefficient corresponding to the frequency f[i], which is the ratio of the target MTF $m_{S}[i]$ to the target MTF $m_{T}[i]$ as follows:

(2) $$c\left[i\right]\;=m_{T}\left[i\right]/m_{S}[i]$$

Here, $c\left[i\right]$ = 1 when both MTFs are equivalent. When $c\left[i\right]>1$, the source MTF is amplified. In contrast, when $c\left[i\right]<1$, the source MTF must be attenuated. $r\left[i\right]$ is the change ratio of the increase or decrease between coefficients *c*[i−1] and $c\left[i\right]$ at one lower frequency f[i−1], defined as $r\left[1\right]=1$. The details of the specific quantization method, initial setting of the change ratio, and method for determining the coefficient array are described in Section 2.2.1, Section 2.2.2 and Section 2.2.3, respectively.

#### 2.2.1. Quantization Method

**Figure 2:**
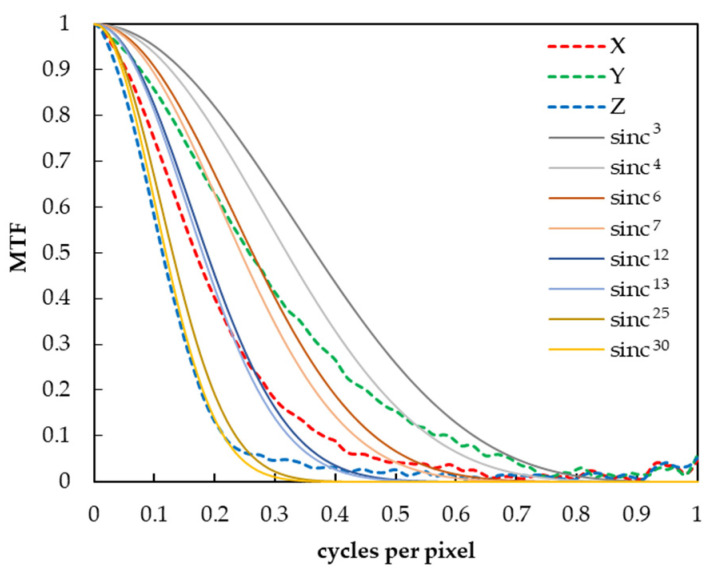
Comparison of MTF of X, Y, and Z images and nth power of sinc function.

In this study, we hypothesized that the MTF follows a sinc function when the pixels of the digital camera are composed of rectangular apertures. To verify this rationality, we first checked the MTF of the CIEXYZ images captured by a 2D spectroradiometer SR-5100 (Topcon Technohouse Corp., Tokyo, Japan) used as the digital camera in this experiment. As a result, it was confirmed that the Y, X, and Z images were approximated to the 6th to 7th, 12th to 13th, and 25th to 30th powers of the sinc function, respectively, as shown in Figure 2. In addition, in preparation for a higher MTF conversion, we assumed that the camera MTF could be approximated in the range of 3 to the 30th power of the sinc function in this experiment, based on a previous study [24] that showed that the general camera MTF can be approximated by the 3rd to 4th powers of the sinc function.

**Figure 3:**
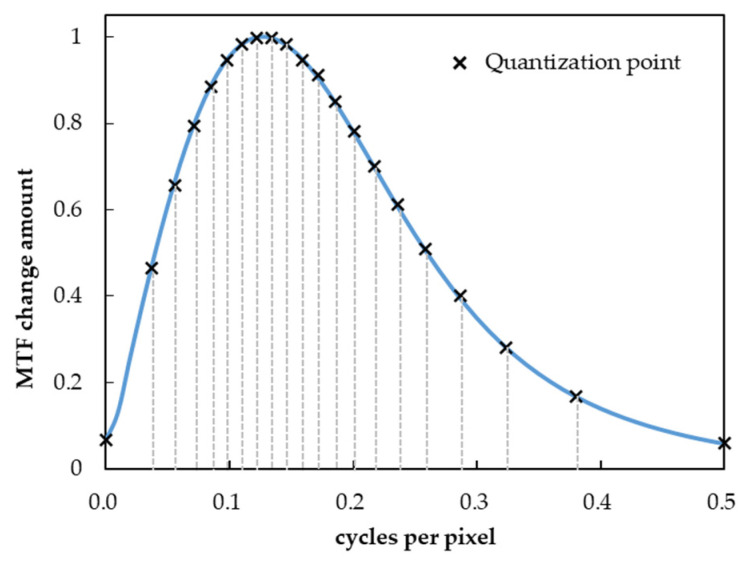
MTF change and quantization point of sinc function.

In the proposed method, to reduce computational complexity, the effective coefficients are obtained by interpolating the effective coefficients for the entire frequency range after quantizing the frequency space to N points in the MTF conversion process. The quantization method is based on the absolute mean value of the MTF change obtained from the derivative of the sinc function to the power of 3 to 30, as assumed above (Figure 3), and quantizes equally for the sum of the changes. In this study, quantization was performed using N = 21 (5% of the steps). By quantizing in this manner to equalize the sum of the change quantization, the frequency band where the MTF changes drastically can be quantized intensively, which has the advantage that the source MTF can be converted efficiently, even with a small number of quantizations.

**Figure 4:**
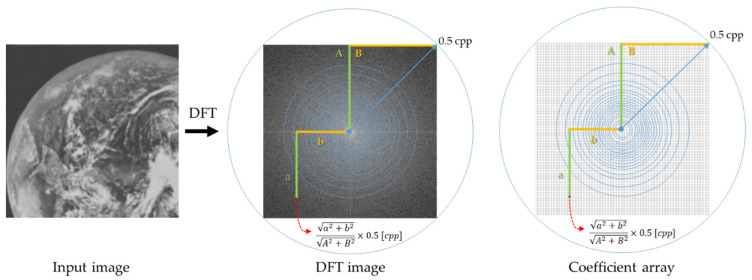
Relationship between the coordinate locations of the input image and cpp in spatial frequency space. The distance from the center to the four corners 0.5 cpp is represented as (A,B), and a specific frequency spectrum is represented as (a,b).

Using the aforementioned method, the quantization points for the frequency at which the change ratio r[i] is calculated are set to 21 points in f[i] = 0, 0.037, 0.056, 0.072, 0.085, 0.098, 0.110, 0.122, 0.134, 0.146, 0.158, 0.172, 0.185, 0.200, 0.217, 0.236, 0.258, 0.286, 0.323, 0.380, 0.500 (i = 1, 2, …, 21) for the interval [0, 0.5] cpp up to the Nyquist frequency. This is fixed regardless of the input image and coefficient array sizes. Figure 4 shows the correspondence between the coordinates of the input image to be converted, coefficient array, and cpp. The coefficient array has a diagonal of 1.0 cpp, with 0.5 cpp from the center to the four corners, and solid concentric circles are drawn for the frequencies of the quantization points. The coefficients of the frequencies other than the quantization point were obtained by linear interpolation from the coefficients of the neighboring quantization points. In other words, the frequency components located in concentric circles in the frequency space have the same coefficients.

#### 2.2.2. Setting the Initial Value of the Change Ratio and Calculating the Effective Coefficient

In this paper, the coefficient c[i] at frequency f[i] is calculated by c[i−1] and the change ratio r[i] according to Equation (1). Therefore, it is necessary to estimate the change ratio r[i] at frequency f[i]. Here, r[i] was obtained through numerical optimization.

First, the initial value of the change ratio ${r[i]}^{\left(0\right)}$ is set as the ratio of the target MTF $m_{T}$ to the converted source MTF $m_{S}$ at adjacent quantization points using the following formula:

(3) $$\begin{array}{c} {r\left[i\right]}^{\left(0\right)}=\frac{m_{T}\left[i\right]}{m_{S}\left[i\right]}/\frac{m_{T}\left[i-1\right]}{m_{S}\left[i-1\right]},\left(i=2,3,\ldots ,N\right)\\ {r[1]}^{\left(0\right)}=1 \end{array}$$

To bring the source MTF closer to the target MTF, the change ratio is numerically optimized by sequential processing from low to high frequencies, such that the mean square error (MSE) between the target MTF and the converted MTF becomes smaller. Details of the MSE calculation method are provided below.

Optimization was performed by obtaining search candidates for change ratios at neighboring quantization points and calculating pairs of change ratios that optimize the MSE by combining pairs of such search candidates. The search candidates are ${r[i]}^{\left(0\right)}\pm \alpha |1-{r[i]}^{\left(0\right)}|$, where α is a parameter that represents the range and interval of the search. For example, if α = 0, 0.5, 0.75, 1.0, 1.25, 1.5, there will be 11 search candidates. The range of change in this search was large for the MTF frequencies, where the relationship before and after a particular frequency significantly increased or decreased. This allowed the variation range to be flexibly defined according to the degree of change.

**Figure 5:**
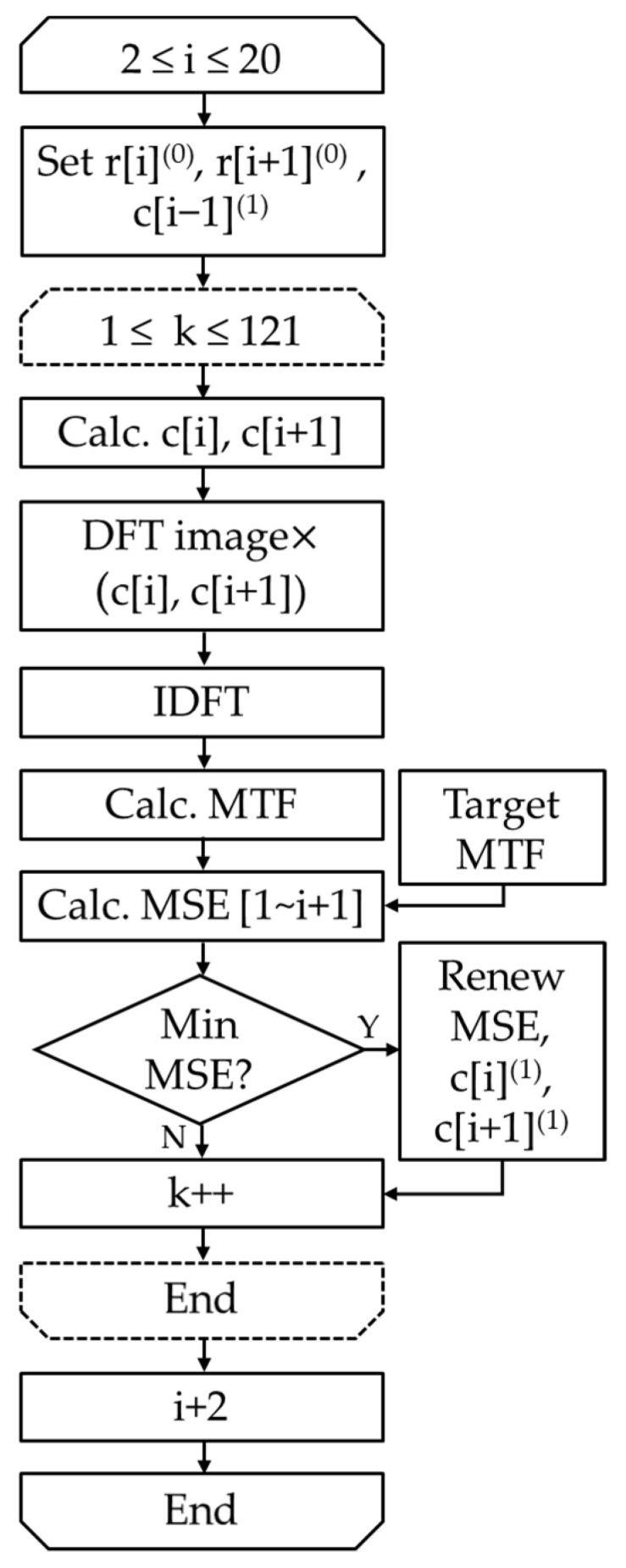
Flowchart of effective coefficient calculation.

The flowchart for calculating the effective coefficients is shown in Figure 5. For each search candidate with a specified change ratio, create a coefficient array consisting of ${r[i]}^{\left(1\right)}$, starting from f[2], calculate the MSE for all candidate change-ratio pairs in adjacent f[i] and f[i+1] on a round-robin basis, and search for the minimum ${r[i]}^{\left(1\right)}$. To calculate ${r[i]}^{\left(1\right)}$, a search is performed for the combination pairs of f[i] and f[i+1] variation candidates; in this study, the number of candidate search pairs is 11 × 11 = 121. This is obtained in the order of i = 2, 3, …, 20 from the low-frequency to the high-frequency side, and the optimal change ratio ${r[i]}^{\left(1\right)}$ is determined in this order. According to the relationship in Equation (1), the coefficient ${c[i]}^{\left(1\right)}$ can be calculated using the coefficient ${c[i-1]}^{\left(1\right)}$ of the previous quantization frequency. This was defined as the effective coefficient. In this case, ${c[1]}^{\left(1\right)}=1$. To further improve the accuracy, this process can be repeated to obtain ${r[i]}^{\left(t+1\right)}$ at the (*t* + 1)th iteration sequentially from ${r[i]}^{\left(t\right)}at\;t$-th iteration.

The effective coefficients for frequencies other than f[i] can be obtained by the linear interpolation of the obtained effective coefficients, and an effective coefficient array for all frequencies can be obtained. After performing the MTF conversion in the frequency space using the created effective coefficient array, an IDFT was performed, and the real part was extracted. This image is the output of the MTF conversion.

#### 2.2.3. MSE Calculation Method

As mentioned above, the change ratio was determined starting from f[2], sequentially from lower frequencies. The MSE was calculated for all change-ratio pairs of search candidates at f[i] and f[i+1] using the following equation:

(4) $$MSE=\int_{f[1]}^{f[N]}{\left(m_{T}\left[f\right]-m_{C}[f]\right)}^{2}df$$

Concretely, c[i] is calculated from the change ratio r[i] and c[i−1], and c[i+1] is calculated from r[i+1] and c[i], according to the relationship in Equation (1). From c[1] to c[i−1], the optimized effective coefficients are used, and from c[i+2] to c[N], which have not yet been optimized, the coefficients are obtained by linear interpolation between adjacent coefficients at 0.001 cpp intervals using coefficients calculated from the initial change ratio of the increase or decrease. The reason for using 0.001 cpp intervals was that the values were stable without significant changes even when the MSE was calculated at intervals finer than 0.001 cpp. The obtained coefficients were multiplied by the spectrum in the frequency space, and the MTF $m_{C}$ is calculated again from the image obtained by the IDFT. For the obtained MTFs, the MSE with the target MTF $m_{T}$ in the interval [f[1], f[N]] was calculated using Equation (4). Find the combination of the change ratio of increase or decrease that minimizes this and makes it the effective coefficient of f[i] and f[i+1].

Similarly, the effective coefficients from f[i+1] to f[i+3] were derived in 0.001 cpp increments. By repeating this until f[N], the effective coefficients can be derived at 0.001 cpp intervals in the range of [0, 0.5] cpp. The accuracy can be improved by using the optimized change ratio ${r[i]}^{\left(t\right)}$ in f[i](i=2, 3,…, N) as the initial value and iteratively updating the optimal change ratio ${r[i]}^{\left(t+1\right)}$ to obtain the effective coefficient ${c[i]}^{\left(t+1\right)}$. In this case, convergence to the optimal MTF can be achieved efficiently by narrowing the search range of α as the number of iterations *t* increases.

## 3. Experiments

Examples of experimental results based on the proposed method are presented in this section. MTF conversion was performed under two conditions: Condition A, in which MTF characteristics differed among multiple channels within an image acquisition device; and Condition B, in which the MTF characteristics possessed by each channel differed among different image acquisition devices. In Condition A, the MTF of the other channels in the same imaging device is used as the target MTF, and in Condition B, the MTF possessed by different imaging devices is used as the target MTF. In this experiment, the MTF was calculated separately using tilted edges based on ISO 12233 [4]. To verify the effectiveness of the proposed method, the MTF conversion results from the application of an unsharp masking filter [18], which is widely used in the image-blur-sharpening process, are shown along with the results of the proposed method, and compared with their results.

Here, we explain the applications of these filters. The MTF conversion using the unsharp masking filter is performed by convolving the following 3 × 3 filters: D. The filter parameter k was obtained for each input image and conversion was performed. To make processing other than filter processing equivalent to the proposed method, MTF conversion is performed by changing k from 0.01 to 9.0 in 0.01 increments to approach the target MTF, and the parameters are determined to minimize the MSE between the target MTF and the converted MTF in the interval from 0 to the Nyquist frequency of 0.5 cpp; thereafter, MTF conversion is performed.

(5) $$D=\left(\begin{array}{ccc} -\frac{1}{9}k & -\frac{1}{9}k & -\frac{1}{9}k\\ -\frac{1}{9}k & 1+\frac{8}{9}k & -\frac{1}{9}k\\ -\frac{1}{9}k & -\frac{1}{9}k & -\frac{1}{9}k \end{array}\right)$$

### 3.1. Condition A: Among Multichannel Images with Different MTFs within an Imaging Device

In ordinary digital color cameras, color images are generated from RGB or CIEXYZ 3-channel images, RGBW 4-channel images, etc. In spectral cameras, color images are generated from multiple channels corresponding to different wavelengths. In other words, there are multichannel images within the imaging equipment, and there are differences in the imaging characteristics that they possess owing to the different wavelength and spatial dependencies among them. Under Condition A, the MTF of a given channel within the same imaging device was used as the target MTF. For example, if the X image has the highest MTF value, the MTF of the X image is used as the target MTF, and the MTF conversion is performed to improve image blurring by increasing the MTF of the Y and Z images of multiple channels with low MTFs.

In this experiment, by targeting different MTFs in the CIEXYZ channels of the SR-5100 used as a digital camera, we set the Y image with the highest MTF value as the target MTF and performed MTF conversion to improve image blurring by increasing the MTF of the X and Z images of multiple channels with low MTFs. Although general consumer cameras use three channels in the RGB space to produce images, the RGB space is a device-dependent color space affected by the color-rendering technology inherent in the camera for color reproduction. Therefore, we used an imaging device capable of generating color images in a device-independent CIEXYZ space.

**Figure 6:**
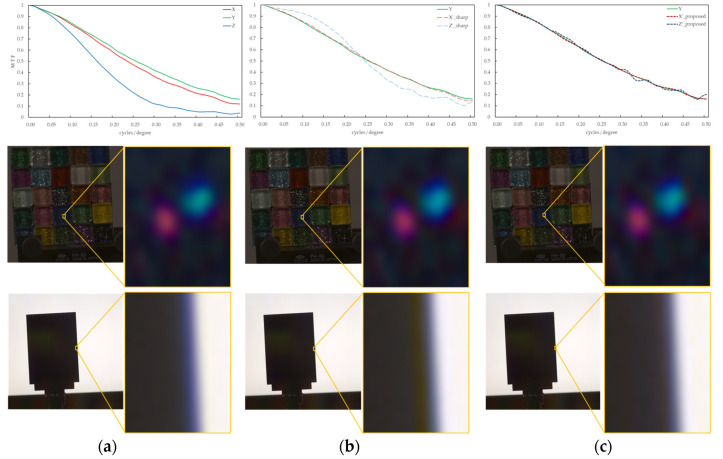
MTF conversion results for multichannel images with different MTFs within an imaging device. (**a**) Input. (**b**) Unsharp masking filter. (**c**) Proposed method.

Figure 6a shows the input images, including an image of colorful glass tiles containing glitter and a slanted black edge illuminated behind it by a plane light source; (b) shows the results of blurring improvement between image channels by the sharpening filter of the conventional method; and (c) shows the conversion results by the proposed method (including the MTF, output image, and partially enlarged image (15 × 20 pixels)). In Figure 6a, the MTF difference between the image channels and the false color of the purple fringes in the image contours are observed. This phenomenon is caused by the wavelength dependency of the image-forming characteristics, such as aberrations in the imaging system, and is an artifact that does not exist in a real system. In the conventional image blur improvement method using an unsharp masking filter shown in Figure 6b, the filter *k* was varied from 0.01 to 0.9 (in 0.01 increments) to find the k with the lowest MSE. The results showed that *k* = 0.17 for the X and *k* = 1.94 for the Z channel. Owing to the sharpening process applied to sharpen the image appearance without considering the optical phenomena, the low-frequency MTF, particularly for the X channel, is excessively and unnaturally increased, and the MTF for the high-frequency channel is significantly reduced toward the high-frequency MTF. In addition, unnaturally distinct reddish contour artifacts are generated near the edges of the image. However, as shown in Figure 6c of the proposed method, such an unnatural contour enhancement did not occur, and the false colors near the edges that occurred in the input image were also reduced, confirming that the blurring of the entire image was reduced. In the image shown in the middle row, colorful glass tiles containing glitter are photographic targets. The proposed method showed a slight improvement in image sharpness, although the difference was not as apparent as that in the edge images.

**Figure 7:**
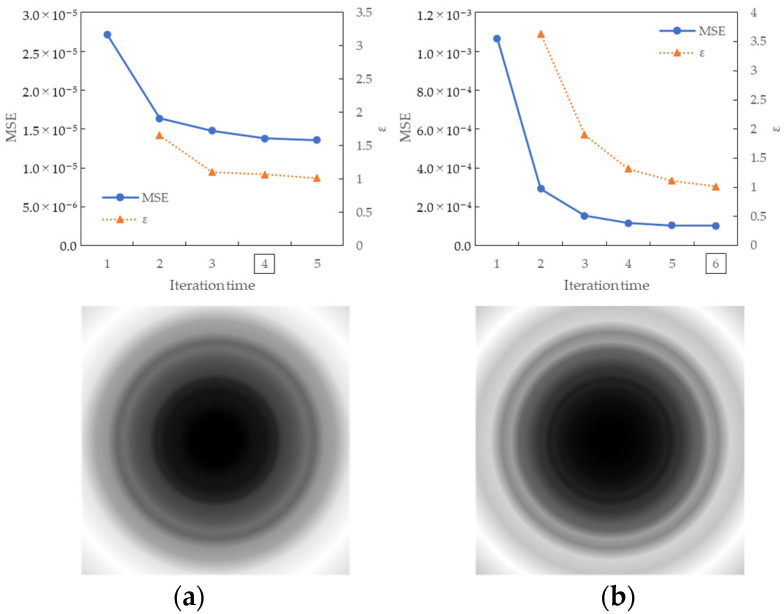
Results of MSE iteration and coefficient array. (**a**) X channel. (**b**) Z channel.

Figure 7 shows the convergence process of the MSE along the iteration times of the MTF conversion and the results of the coefficient array. The convergence condition for MSE was defined as the ratio ε of the current MSE and MSE in the previous iteration. When ε≤1.1 was satisfied, the iteration was terminated and considered convergence. In the figure, the number of convergence iterations is indicated by a square, and convergence is achieved after four and six iterations for the X and Z channels, respectively. The coefficient array was visualized in grayscale, with the maximum and minimum values within each channel shown in white and black, respectively. For the X channel, the maximum and minimum values were 1.33 and 1.00 for the X channel and 5.75 and 1.00 for the Z channel. In particular, enhancement was achieved in the high-frequency band, and the Z channel of the input with a low MTF was significantly improved. It was confirmed that the proposed method could approximate the target MTF in the MTF conversion for multiple channels with different MTFs in the imaging equipment.

### 3.2. Condition B: Among Image Channels with different MTFs between Different Imaging Devices

Widely used imaging devices generally have different image formation characteristics owing to their different designs, such as optical systems and circuits. Therefore, when image formation characteristics are compared between multiple imaging devices, images with different appearances, such as blurring, are acquired and generated. In Condition B, the MTF conversion is performed using different MTFs of the different imaging devices as the target MTF. In the experiment, the same camera SR-5100 as in Condition A was used in the out-of-focus condition to simulate an imaging device with inferior image formation characteristics to compare different image formation characteristics (otherwise the same conditions). Thus, the MTF of each CIEXYZ channel is closer to the target MTF. For example, an experiment is conducted in which the target MTF is set to the higher MTF owned by a different imaging device, and the MTF of the channels of the imaging device with low MTF is converted to be closer to the target MTF.

**Figure 8:**
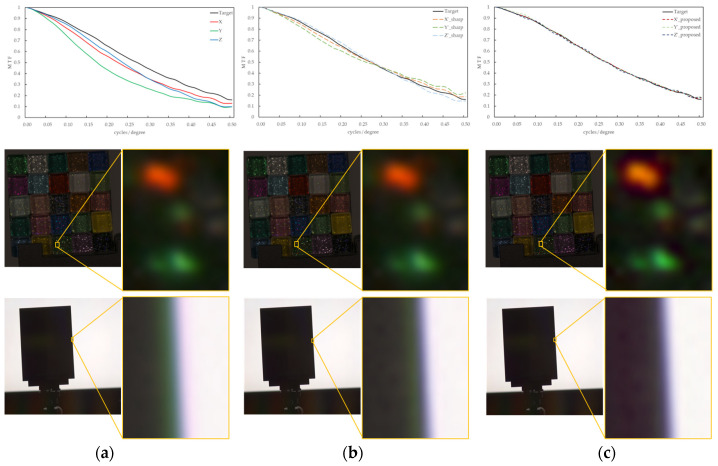
MTF conversion results for image channels with different MTFs between different imaging devices. (**a**) Input. (**b**) Unsharp masking filter. (**c**) Proposed method.

Figure 8 shows an example of the MTF conversion assuming between imaging devices with different image-forming characteristics for an image taken of the same objects as in Condition A, such as a colored glitter glass image and an edge image. As in Condition A, three image channels (X, Y, and Z) were used to avoid issues such as those caused by tone mapping from CIEXYZ to the RGB space. From Figure 8a, the MTF difference between the image channels and the occurrence of a green false color in the image contours generated from them were confirmed. This artifact did not exist in the real object, as shown in Figure 7a. The k results for the unsharp masking filter shown in Figure 8b were 0.31, 0.84, 0.30 for the X, Y, and Z channels, respectively. Green artifacts are clearly visible near the black edges of the edge image. By comparing Figure 8b,c, it is confirmed that the proposed method is closer to the target MTF and produces a natural and clear appearance of the edge contours in the image. In addition, in the enlarged image of the glitter in the glass tile, the highlight of the glitter appeared to be more prominent in the proposed method than in the blurred input image.

**Figure 9:**
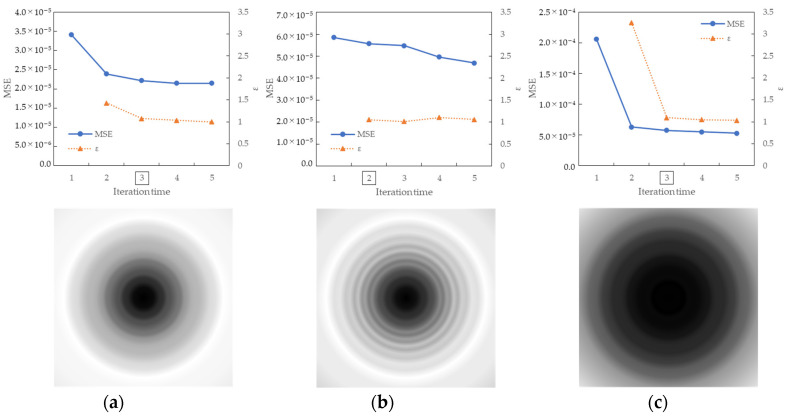
Results of MSE iteration and coefficient array. (**a**) X channel. (**b**) Y channel. (**c**) Z channel.

Figure 9 shows the results of the MSE iteration and coefficient array. As in Condition A, iterations were performed until MSE with ε ≤ 1.1, and the iteration times of convergence were 3, 2, and 3 for the X, Y, and Z channels, respectively. The maximum and minimum values of the coefficient array were max 1.30, min 1.00 for X, max 1.83, min 1.00 for Y, and max 1.98, min 1.00 for Z channel. The number of iterations required for convergence was small, confirming that the MTF conversion results accurately approximated the target MTF.

### 3.3. Discussion and Issues

Compared to the conventional unsharp masking method for improving image blurring related to the spatial resolution of the image, MTF conversion using the proposed method can achieve natural image processing with no false color or edge enhancement, following the MTF allowed by the camera MTF. Table 1 summarizes the results of obtaining the SSIM and PNSR values for the Y channel based on the following: (i) is an image captured by an actual camera with the target MTF; (ii) is an image with only a different MTF compared to (i) (conditions other than focus are the same); (iii) is an image obtained by taking (ii) as the input image and converting it using the proposed method to obtain the MTF of (i); and (iv) is an image obtained by taking (ii) as the input image and performing MTF conversion using the sharpening filter. Each image shows a close-up view of an edge image. The SSIM and PSNR values between each image in (ii)–(iv) and that in (i) were calculated, and the improvement in the values was confirmed by the proposed method and sharpening filter, indicating that the proposed method was more effective than the sharpening filter.

However, several issues remain to be resolved. Our proposed method targets the conversion of interchannel or interdevice characteristics. Therefore, the amount of conversion is small compared to the restoration of the degraded image like the deconvolution algorithm, which transforms it into an ideal image. Our method performs frequency transformations, which theoretically creates the problem of noise enhancement; however, the effect is sufficiently small to be negligible because of the small number of target transformations, and we do not include regularization or any other smoothing of the image space as a constraint. Therefore, the MSE error has the advantage of being extremely small because it can be adjusted purely based on the MTF difference. For conversions with significant MTF differences, the proposed method may not be applicable because of the emphasis on noise, and there may be a limitation in the proposed method. For the purpose of this study, the situation is so small that its influence is negligible owing to the small number of targeted conversions. The MTF conversion affects the frequency characteristics of the image; therefore, a careful approach is required for conversion.

To solve the issue of improving color reproduction, it is necessary to perform MTF conversion in the CIEXYZ space, which is independent of the imaging device, and to consider the correspondence between the RGB space, which is independent of the imaging device, and the CIEXYZ space, which is independent of the imaging device, by considering again, because the rendering method inside the imaging device is a black box. For future development, it is necessary to explore image reproduction methods that consider their relationship with color reproduction. As a preliminary step in the experiments described in our paper, we validated our results using images with different numbers of channels (CIEXYZ images and spectral images every 1 nm). Although the accuracy could be improved by dividing the visible light wavelength range into more groups, based on our experimental conditions, we concluded that there was no need to increase the number of channels for the following three reasons: (1) The human eye could not recognize any difference between the results of the converted spectral and CIEXYZ images; (2) the improvement in accuracy was not expected to be sufficient to be worth the computational workload; and (3) because spectral images were converted to CIEXYZ images in the subsequent step, this was more efficient, and the MSE accuracy of the condition for the three CIEXYZ channels was sufficiently small.

Regarding the computational load, it is necessary to consider measures such as adjusting the number of parameters in steps according to the desired accuracy based on the tradeoff between the coefficient calculation and the desired MTF conversion accuracy. In this study, we employed a method to improve approximation accuracy by sampling at 5% steps with respect to the sum of the change ratios, dividing the frequency band from low to high into 20 bands, and obtaining effective coefficients. The more quantization points are added, the more MSE reduction and MTF approximation accuracy can be achieved. Therefore, there is still potential for further study, together with the acceleration of the MTF conversion algorithm. In addition, it is necessary to address the side effects of MTF conversion, such as an increase in noise and the amount of computation that occurs as a result of MTF improvement.

In this study, the MTF was calculated using the ISO 12233 edge method. However, many studies have discussed speeding up the MTF calculation method, and future research on speeding up the MTF calculation and improving its accuracy by introducing these methods as appropriate is required. In the proposed method, the iterative process to converge the MSE value was adopted as a starting point. However, other methods, such as the goodness-of-fit coefficient, should also be considered in the MTF approximation method to explore better approaches from the viewpoints of accuracy and speed. These are important research questions for improving image-processing and optical technologies.

It is expected that the results of this study can be used to solve various image-forming characteristic issues by implementing an MTF conversion that considers the differences in image-forming characteristics among common imaging devices. In addition, because the MTF conversion target can be measured or simulated using existing methods, it has the potential to contribute to the development of various industrial technologies such as reducing the cost of prototypes by applying image simulation technology to the design of imaging equipment. This is applicable not only to image-capturing devices such as cameras, as shown in this study, but is also expected to be applicable to image-generating devices such as displays.

## 4. Conclusions

In this study, we proposed a method to address the issue of differing characteristics that inevitably rise within or between imaging devices. We focused on the characteristics of MTF, an optical performance index that allows them to be converted automatically among image channels within or between imaging devices. The results of the MTF conversion for multichannel images with different image-forming characteristics within an imaging device showed that it is possible to generate sharper images by approximating the target MTF. The physical resolution characteristics can be controlled by the MTF conversion enabled in our study, and as a future step, we will begin to develop control techniques that consider perceptual resolution.

We believe that this study is important because the following technical and academic progress is expected by converting the MTF between image channels using the proposed method, which has not yet been addressed. (1) Even if the imaging characteristics of the hardware are unknown, the MTF can be converted to the target MTF using the image after it is captured. (2) As any MTF can be converted into a target, image simulation for conversion to a different MTF is possible. (3) It is possible to generate high-definition images, thereby meeting the requirements of various industrial and research fields in which high-definition images are required. This study is expected to address various image-forming characteristic issues, potentially leading to the advancement of various industrial technologies. For example, it has the potential to reduce prototype costs through the application of image simulation technology in the design of imaging equipment. Our method may be applied not only to imaging devices such as cameras but also to image-producing devices such as displays. Consequently, this study is expected to serve as a foundational technology for comprehensively improving the image reproduction capabilities of imaging devices.

## 5. Patents

Patents related to the results reported in this manuscript are currently being processed.

## Figures and Tables

**Table 1:** Quantitative comparison using SSIM and PSNR.

(i) Ideal Image	(ii) Blurred Image	(iii) Proposed Method	(iv) Sharpening Filter
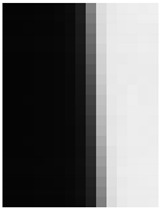	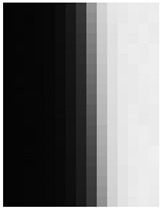	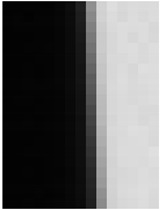	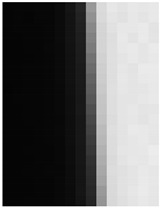
-	SSIM = 0.911	SSIM = 0.983	SSIM = 0.959
-	PSNR = 28.19 [dB]	PSNR = 31.23 [dB]	PSNR = 30.96 [dB]

## Data Availability

Dataset available on request from the authors.
